# Supplementary material for: Impact of Adding GLP-1 Receptor Agonists to Insulin Therapy on Cardiovascular and Microvascular Outcomes in Type 2 Diabetes: A Nationwide Cohort Study from Taiwan
Source: Pharmaceuticals (Basel). 2025 Sep 12;18(9):1368. doi: 10.3390/ph18091368 (PMC12472429; doi:10.3390/ph18091368)
Supplement: Supplementary file 1 [file pharmaceuticals-18-01368-s001.zip › pharmaceuticals-3825767-supplementary.pdf]

Table S1 :Characteristics of patients with T2D treated with insulin and sulfonylurea or GLP-1 RA

Variable	Sulfonylurea users (N=5242)		GLP-1 RA users (N=5242)		SMD
	n	%	n	%	
Sex					0.006
Female	2534	48.34	2551	48.66	
Male	2708	51.66	2691	51.34	
Age, years					0.028
20-60	3653	69.69	3719	70.95	
61-100	1589	30.31	1523	29.05	
Mean ± SD	52.85	13.28	52.46	13.37	0.029
Comorbidities					
Obesity	512	9.77	587	11.20	0.047
Smoking	262	5.00	274	5.23	0.010
Alcohol-related disorders	152	2.90	148	2.82	0.005
Hypertension	3576	68.22	3534	67.42	0.017
Dyslipidemia	4431	84.53	4347	82.93	0.043
Coronary artery disease	815	15.55	811	15.47	0.002
Stroke	381	7.27	385	7.34	0.003
Heart failure	128	2.44	123	2.35	0.006
Atrial fibrillation	348	6.64	353	6.73	0.004
Peripheral artery disease	211	4.03	203	3.87	0.008
COPD	494	9.42	488	9.31	0.004
Cirrhosis	111	2.12	104	1.98	0.009
Chronic kidney disease	668	12.74	679	12.95	0.006
Retinopathy	1339	25.54	1388	26.48	0.021
Cancers	276	5.27	268	5.11	0.007
Charlson Comorbidity Index					0.023
≤1	4060	77.45	4111	78.42	
>1	1182	22.55	1131	21.58	
Diabetes Complications Severity Index					0.010
≤1	2685	51.22	2710	51.70	
>1	2557	48.78	2532	48.30	
Medications					
DPP-4 inhibitors	4154	79.24	4088	77.99	0.031
SGLT2 inhibitors	1444	27.55	1576	30.06	0.056
Thiazolidinedione	2337	44.58	2311	44.09	0.010

Alpha-glucosidase inhibitor	2265	43.21	2253	42.98	0.005
Metformin	5050	96.34	5008	95.54	0.041
Premix insulins	1626	31.02	1621	30.92	0.002
Short insulins	2456	46.85	2419	46.15	0.014
Basal insulins	4523	86.28	4484	85.54	0.021
ACEI	1478	28.20	1461	27.87	0.007
ARB	3183	60.72	3158	60.24	0.010
α-Blockers	460	8.78	446	8.51	0.010
β-blocker	2613	49.85	2635	50.27	0.008
Calcium-channel blockers	3000	57.23	2930	55.89	0.027
Diuretic	1992	38.00	1952	37.24	0.016
Statin	3997	76.25	3957	75.49	0.018
Aspirin	2355	44.93	2325	44.35	0.012
Number of oral antidiabetic drugs					0.031
≤3	1241	23.67	1310	24.99	
>3	4001	76.33	3932	75.01	
Duration of T2D, years					0.017
≤5	1587	30.27	1546	29.49	
>5	3655	69.73	3696	70.51	
Mean ± SD	7.17	3.80	7.11	3.72	0.015

Abbreviations: T2D = type 2 diabetes; GLP-1 RA = glucagon-like peptide-1 receptor agonists; SD = standard deviation; COPD = chronic obstructive pulmonary disease; DPP-4 = Dipeptidyl peptidase-4; SGLT-2 = sodium glucose cotransporter-2; ACEI = angiotensin-converting enzyme inhibitor; ARB = angiotensin receptor blocker.

*A standard mean difference (SMD) < 0.1 indicates a negligible difference between GLP-1 RA and sulfonylurea users.

Table S2: Outcome risks of adding DPP-4 inhibitors versus GLP-1 RA to insulin therapy in patients with type 2 diabetes, excluding SGLT2 inhibitor users

Outcome	DPP-4 inhibitor users			GLP-1 RA users			cHR	(95% CI)	aHR†	(95% CI)
	n	PY	IR	n	PY	IR				
Primary outcomes										
Major adverse cardiovascular events[a]	613	16984	36.09	345	17742	19.45	0.54	(0.47, 0.61)***	0.51	(0.45, 0.59)***
Major microvascular outcomes[b]	330	17654	18.69	131	18235	7.18	0.39	(0.32, 0.48)***	0.41	(0.33, 0.50)***
Secondary outcomes										
Hospitalization for coronary artery disease	297	17737	16.74	189	18102	10.44	0.62	(0.52, 0.74)***	0.6	(0.50, 0.72)***
Hospitalization for stroke	313	17776	17.61	159	18202	8.74	0.49	(0.41, 0.60)***	0.48	(0.40, 0.58)***
Hospitalization for heart failure	191	18042	10.59	61	18432	3.31	0.32	(0.24, 0.42)***	0.31	(0.23, 0.42)***
End-stage kidney disease	137	18188	7.53	9	18539	0.49	0.07	(0.03, 0.13)***	0.07	(0.04, 0.14)***
Sight-threatening retinopathy	200	17916	11.16	120	18253	6.57	0.59	(0.47, 0.74)***	0.61	(0.49, 0.77)***
Leg amputation			0.87			0.11	0.13	(0.03, 0.59)**	0.15	(0.03, 0.68)*
All-cause mortality	419	18475	22.68	185	18551	9.97	0.44	(0.37, 0.53)***	0.4	(0.33, 0.48)***

Abbreviations: DPP-4 = dipeptidyl peptidase-4; GLP-1 RA = glucagon-like peptide-1 receptor agonists; SGLT-2 = sodium glucose cotransporter-2; PY = Person-Year; IR = incidence rate per 1,000 person-years; cHR = crude hazard ratio; aHR = adjusted hazard ratio; CI = confidence interval.

† aHR adjusted for age, gender, comorbidities, CCI, DCSI, concomitant medications, and duration of T2D, as detailed in Table 1.

[a] Composite outcome includes hospitalizations for coronary artery disease, stroke, and heart failure.

[b] Composite outcome includes end-stage kidney disease, sight-threatening retinopathy, and non-traumatic leg amputation.

* $p < 0.05$, ** $p < 0.01$, *** $p < 0.001$.

Table S3: Hospitalization for CAD Risk, Stratified by Variables, in Insulin-treated T2D Patients on GLP-1 RA vs. DPP-4 Inhibitors

	DPP-4 inhibitor users			GLP-1 RA users						
Variables	n	PY	IR	n	PY	IR	cHR	(95% CI)	aHR	(95% CI)
Sex										
Female	146	10755	13.58	86	11529	7.46	0.55	(0.42, 0.72)***	0.55	(0.42, 0.72)***
Male	213	11562	18.42	172	12558	13.70	0.73	(0.60, 0.90)**	0.69	(0.56, 0.85)***
Age, years										
20-60	233	17352	13.43	170	18577	9.15	0.68	(0.56, 0.83)***	0.70	(0.58, 0.86)***
61-100	126	4965	25.38	88	5509	15.97	0.63	(0.48, 0.83)***	0.57	(0.43, 0.76)***
Hypertension										
No	65	7763	8.37	59	8193	7.20	0.86	(0.61, 1.23)	0.81	(0.57, 1.17)
Yes	294	14554	20.20	199	15893	12.52	0.62	(0.51, 0.74)***	0.59	(0.49, 0.71)***
Dyslipidemia										
No	46	3483	13.21	24	3938	6.09	0.46	(0.28, 0.75)**	0.41	(0.25, 0.69)***
Yes	313	18833	16.62	234	20148	11.61	0.70	(0.59, 0.83)***	0.67	(0.56, 0.79)***
CCI										
≤1	267	18487	14.44	201	19699	10.20	0.70	(0.58, 0.84)***	0.68	(0.56, 0.82)***
>1	92	3830	24.02	57	4388	12.99	0.54	(0.39, 0.75)***	0.53	(0.38, 0.75)***
DCSI										
≤1	122	12505	9.76	89	13472	6.61	0.67	(0.51, 0.88)**	0.66	(0.50, 0.87)**
>1	237	9812	24.15	169	10614	15.92	0.66	(0.54, 0.81)***	0.64	(0.52, 0.78)***
Number of oral antidiabetic drugs										

0-3	59	4975	11.86	51	5707	8.94	0.75	(0.52, 1.09)	0.77	(0.52, 1.13)
>3	300	17342	17.30	207	18379	11.26	0.65	(0.54, 0.77)***	0.62	(0.52, 0.74)***
Statin										
No	62	5208	11.90	49	5979	8.19	0.68	(0.47, 0.99)*	0.59	(0.40, 0.87)**
Yes	297	17109	17.36	209	18107	11.54	0.66	(0.56, 0.79)***	0.65	(0.54, 0.78)***
Duration of T2D, years										
≤5	88	7705	11.42	57	7806	7.30	0.65	(0.46, 0.90)*	0.74	(0.52, 1.04)
>5	271	14612	18.55	201	16280	12.35	0.66	(0.55, 0.79)***	0.63	(0.52, 0.75)***

Abbreviations: CAD, coronary artery disease; T2D, type 2 diabetes; GLP-1 RA, glucagon-like peptide-1 receptor agonists; DPP-4, dipeptidyl peptidase-4; PY: person-year, IR: incidence rate, per 1000 persons/years; cHR: crude hazard ratio; aHR: adjusted hazard ratio; CI: confidence interval. COPD, chronic obstructive pulmonary disease; CCI, Charlson Comorbidity Index; DCSI, Diabetes Complication Severity Index.

aHR: adjusted for age, sex, obesity, comorbidities, CCI, DCSI scores, medications, and duration of T2D as listed in Table 1 using the Cox proportional hazards regression. * p<0.05, ** p<0.01, *** p<0.001.

Table S4: Hospitalization for stroke Risk, Stratified by Variables, in Insulin-treated T2D Patients on GLP-1 RA vs. DPP-4 Inhibitors

	DPP-4 inhibitor users			GLP-1 RA users						
Variables	n	PY	IR	n	PY	IR	cHR	(95% CI)	aHR	(95% CI)
Sex										
Female	195	10663	18.29	100	11501	8.70	0.47	(0.37, 0.60)***	0.48	(0.38, 0.61)***
Male	205	11648	17.60	112	12721	8.80	0.50	(0.40, 0.63)***	0.47	(0.37, 0.60)***
Age, years										
20-60	247	17368	14.22	126	18684	6.74	0.47	(0.38, 0.59)***	0.48	(0.39, 0.60)***
61-100	153	4943	30.95	86	5537	15.53	0.51	(0.39, 0.66)***	0.46	(0.35, 0.61)***
Hypertension										
No	68	7778	8.74	45	8233	5.47	0.62	(0.43, 0.91)*	0.61	(0.42, 0.90)*
Yes	332	14533	22.84	167	15988	10.45	0.46	(0.38, 0.55)***	0.45	(0.37, 0.54)***
Dyslipidemia										
No	56	3480	16.09	34	3918	8.68	0.53	(0.34, 0.81)**	0.53	(0.34, 0.82)**
Yes	344	18830	18.27	178	20303	8.77	0.48	(0.40, 0.58)***	0.47	(0.39, 0.56)***
CCI										
≤1	274	18528	14.79	162	19822	8.17	0.55	(0.45, 0.67)***	0.55	(0.45, 0.67)***
>1	126	3783	33.31	50	4400	11.36	0.35	(0.25, 0.48)***	0.32	(0.23, 0.45)***
DCSI										
≤1	145	12471	11.63	93	13478	6.90	0.59	(0.46, 0.77)***	0.59	(0.45, 0.77)***
>1	255	9840	25.92	119	10744	11.08	0.43	(0.34, 0.53)***	0.41	(0.33, 0.52)***
Number of oral antidiabetic drugs										

0-3	53	4982	10.64	39	5725	6.81	0.64	(0.42, 0.97)*	0.58	(0.38, 0.88)*
>3	347	17329	20.02	173	18496	9.35	0.47	(0.39, 0.56)***	0.46	(0.38, 0.55)***
Statin										
No	81	5193	15.60	51	5988	8.52	0.55	(0.39, 0.78)***	0.53	(0.37, 0.76)***
Yes	319	17118	18.64	161	18233	8.83	0.47	(0.39, 0.57)***	0.47	(0.38, 0.56)***
Duration of T2D, years										
≤5	113	7687	14.70	54	7824	6.90	0.48	(0.35, 0.67)***	0.49	(0.35, 0.68)***
>5	287	14624	19.63	158	16397	9.64	0.49	(0.40, 0.59)***	0.47	(0.39, 0.57)***

Abbreviations: T2D, type 2 diabetes; GLP-1 RA, glucagon-like peptide-1 receptor agonists; DPP-4, dipeptidyl peptidase-4; PY: person-year, IR: incidence rate, per 1000 persons/years; cHR: crude hazard ratio; aHR: adjusted hazard ratio; CI: confidence interval. COPD, chronic obstructive pulmonary disease; CCI, Charlson Comorbidity Index; DCSI, Diabetes Complication Severity Index.

aHR: adjusted for age, sex, obesity, comorbidities, CCI, DCSI scores, medications, and duration of T2D as listed in Table 1 using the Cox proportional hazards regression. * p<0.05, ** p<0.01, *** p<0.001.

Table S5: Hospitalization for heart failure Risk, Stratified by Variables, in Insulin-treated T2D Patients on GLP-1 RA vs. DPP-4 Inhibitors

	DPP-4 inhibitor users			GLP-1 RA users						
Variables	n	PY	IR	n	PY	IR	cHR	(95% CI)	aHR	(95% CI)
Sex										
Female	108	10842	9.96	36	11663	3.09	0.31	(0.22, 0.46)***	0.32	(0.22, 0.48)***
Male	123	11813	10.41	45	12845	3.50	0.34	(0.24, 0.48)***	0.33	(0.23, 0.47)***
Age, years										
20-60	153	17574	8.71	46	18886	2.44	0.28	(0.20, 0.40)***	0.31	(0.22, 0.43)***
61-100	78	5082	15.35	35	5622	6.23	0.41	(0.27, 0.61)***	0.36	(0.24, 0.54)***
Hypertension										
No	51	7830	6.51	15	8292	1.81	0.27	(0.15, 0.48)***	0.26	(0.14, 0.46)***
Yes	180	14826	12.14	66	16217	4.07	0.34	(0.26, 0.45)***	0.34	(0.25, 0.45)***
Dyslipidemia										
No	39	3513	11.10	11	3969	2.77	0.25	(0.13, 0.48)***	0.22	(0.11, 0.44)***
Yes	192	19143	10.03	70	20540	3.41	0.35	(0.26, 0.46)***	0.35	(0.26, 0.46)***
CCI										
≤1	167	18772	8.90	67	20033	3.34	0.38	(0.29, 0.51)***	0.37	(0.28, 0.49)***
>1	64	3884	16.48	14	4475	3.13	0.19	(0.11, 0.34)***	0.23	(0.12, 0.41)***
DCSI										
≤1	65	12665	5.13	22	13632	1.61	0.32	(0.19, 0.51)***	0.32	(0.20, 0.52)***
>1	166	9991	16.62	59	10876	5.42	0.33	(0.25, 0.45)***	0.33	(0.25, 0.45)***
Number of oral antidiabetic drugs										

0-3	30	5042	5.95	15	5780	2.59	0.45	(0.24, 0.83)*	0.40	(0.21, 0.76)**
>3	201	17614	11.41	66	18728	3.52	0.31	(0.24, 0.41)***	0.31	(0.23, 0.41)***
Statin										
No	51	5254	9.71	15	6059	2.48	0.26	(0.14, 0.46)***	0.25	(0.14, 0.45)***
Yes	180	17401	10.34	66	18449	3.58	0.35	(0.26, 0.47)***	0.35	(0.26, 0.47)***
Duration of T2D, years										
≤5	59	7799	7.57	16	7913	2.02	0.28	(0.16, 0.48)***	0.29	(0.17, 0.52)***
>5	172	14857	11.58	65	16595	3.92	0.34	(0.26, 0.45)***	0.33	(0.25, 0.44)***

Abbreviations: T2D, type 2 diabetes; GLP-1 RA, glucagon-like peptide-1 receptor agonists; DPP-4, dipeptidyl peptidase-4; PY: person-year, IR: incidence rate, per 1000 persons/years; cHR: crude hazard ratio; aHR: adjusted hazard ratio; CI: confidence interval. COPD, chronic obstructive pulmonary disease; CCI, Charlson Comorbidity Index; DCSI, Diabetes Complication Severity Index.

aHR: adjusted for age, sex, obesity, comorbidities, CCI, DCSI scores, medications, and duration of T2D as listed in Table 1 using the Cox proportional hazards regression. * p<0.05, ** p<0.01, *** p<0.001.

Table S6: ESKD Risk, Stratified by Variables, in Insulin-treated T2D Patients on GLP-1 RA vs. DPP-4 Inhibitors

	DPP-4 inhibitor users			GLP-1 RA users						
Variables	n	PY	IR	n	PY	IR	cHR	(95% CI)	aHR	(95% CI)
Sex										
Female	72	10902	6.60			0.09	0.01	(0.00, 0.10)***	0.02	(0.00, 0.11)***
Male	86	11924	7.21			0.85	0.12	(0.06, 0.23)***	0.12	(0.06, 0.22)***
Age, years										
20-60	119	17667	6.74	5	18951	0.26	0.04	(0.02, 0.10)***	0.05	(0.02, 0.11)***
61-100	39	5159	7.56	7	5684	1.23	0.17	(0.07, 0.37)***	0.12	(0.05, 0.28)***
Hypertension										
No	18	7885	2.28			0.24	0.12	(0.03, 0.50)**	0.10	(0.02, 0.45)**
Yes	140	14941	9.37			0.61	0.07	(0.04, 0.13)***	0.07	(0.04, 0.14)***
Dyslipidemia										
No	24	3552	6.76			0.75	0.11	(0.03, 0.38)***	0.05	(0.01, 0.22)***
Yes	134	19274	6.95			0.44	0.07	(0.03, 0.13)***	0.07	(0.04, 0.14)***
CCI										
≤1	99	18925	5.23	4	20147	0.20	0.04	(0.01, 0.11)***	0.04	(0.01, 0.11)***
>1	59	3901	15.12	8	4487	1.78	0.12	(0.06, 0.25)***	0.15	(0.07, 0.32)***
DCSI										
≤1	33	12729	2.59			0.07	0.03	(0.00, 0.22)***	0.03	(0.00, 0.21)***
>1	125	10097	12.38			1.00	0.08	(0.05, 0.16)***	0.09	(0.05, 0.17)***
Number of oral antidiabetic drugs										

0-3	20	5064	3.95	0.17	0.04	(0.01, 0.32)**	0.05	(0.01, 0.37)**
>3	138	17762	7.77	0.58	0.08	(0.04, 0.14)***	0.08	(0.04, 0.15)***
Statin								
No	31	5312	5.84	0.33	0.06	(0.01, 0.25)***	0.04	(0.01, 0.17)***
Yes	127	17514	7.25	0.54	0.08	(0.04, 0.15)***	0.09	(0.05, 0.17)***
Duration of T2D, years								
≤5	33	7869	4.19	0.25	0.07	(0.02, 0.30)***	0.08	(0.02, 0.35)***
>5	125	14957	8.36	0.60	0.07	(0.04, 0.14)***	0.08	(0.04, 0.15)***

Abbreviations: ESKD, end-stage kidney disease; T2D, type 2 diabetes; GLP-1 RA, glucagon-like peptide-1 receptor agonists; DPP-4, dipeptidyl peptidase-4; PY: person-year, IR: incidence rate, per 1000 persons/years; cHR: crude hazard ratio; aHR: adjusted hazard ratio; CI: confidence interval. COPD, chronic obstructive pulmonary disease; CCI, Charlson Comorbidity Index; DCSI, Diabetes Complication Severity Index.

aHR: adjusted for age, sex, obesity, comorbidities, CCI, DCSI scores, medications, and duration of T2D as listed in Table 1 using the Cox proportional hazards regression. ** p<0.01, *** p<0.001.

Table S7: Sight-threatening retinopathy Risk, Stratified by Variables, in Insulin-treated T2D Patients on GLP-1 RA vs. DPP-4 Inhibitors

	DPP-4 inhibitor users			GLP-1 RA users						
Variables	n	PY	IR	n	PY	IR	cHR	(95% CI)	aHR	(95% CI)
Sex										
Female	105	10776	9.74	74	11535	6.42	0.66	(0.49, 0.89)**	0.67	(0.50, 0.91)**
Male	129	11761	10.97	76	12773	5.95	0.54	(0.41, 0.72)***	0.57	(0.43, 0.76)***
Age, years										
20-60	193	17410	11.09	107	18708	5.72	0.52	(0.41, 0.66)***	0.55	(0.43, 0.70)***
61-100	41	5128	8.00	43	5600	7.68	0.96	(0.62, 1.48)	0.94	(0.61, 1.46)
Hypertension										
No	86	7708	11.16	52	8200	6.34	0.57	(0.41, 0.81)**	0.59	(0.42, 0.84)**
Yes	148	14830	9.98	98	16108	6.08	0.61	(0.47, 0.79)***	0.65	(0.50, 0.84)**
Dyslipidemia										
No	53	3460	15.32	19	3952	4.81	0.32	(0.19, 0.54)***	0.34	(0.20, 0.58)***
Yes	181	19077	9.49	131	20356	6.44	0.68	(0.54, 0.85)***	0.70	(0.56, 0.88)**
CCI										
≤1	188	18624	10.09	122	19878	6.14	0.61	(0.48, 0.77)***	0.62	(0.49, 0.78)***
>1	46	3914	11.75	28	4430	6.32	0.54	(0.34, 0.87)*	0.62	(0.39, 1.01)
DCSI										
≤1	106	12512	8.47	64	13527	4.73	0.57	(0.42, 0.77)***	0.58	(0.43, 0.79)***
>1	128	10026	12.77	86	10781	7.98	0.61	(0.47, 0.81)***	0.64	(0.48, 0.84)**
Number of oral antidiabetic drugs										

0-3	48	4997	9.61	26	5733	4.54	0.48	(0.30, 0.78)**	0.45	(0.28, 0.74)**
>3	186	17540	10.60	124	18575	6.68	0.63	(0.50, 0.79)***	0.66	(0.52, 0.83)***
Statin										
No	69	5201	13.27	35	6023	5.81	0.45	(0.30, 0.67)***	0.48	(0.32, 0.73)***
Yes	165	17336	9.52	115	18285	6.29	0.66	(0.52, 0.84)***	0.68	(0.54, 0.87)**
Duration of T2D, years										
≤5	56	7752	7.22	38	7857	4.84	0.68	(0.45, 1.03)	0.77	(0.50, 1.17)
>5	178	14786	12.04	112	16451	6.81	0.57	(0.45, 0.72)***	0.59	(0.46, 0.74)***

Abbreviations: T2D, type 2 diabetes; GLP-1 RA, glucagon-like peptide-1 receptor agonists; DPP-4, dipeptidyl peptidase-4; PY: person-year, IR: incidence rate, per 1000 persons/years; cHR: crude hazard ratio; aHR: adjusted hazard ratio; CI: confidence interval. COPD, chronic obstructive pulmonary disease; CCI, Charlson Comorbidity Index; DCSI, Diabetes Complication Severity Index.

aHR: adjusted for age, sex, obesity, comorbidities, CCI, DCSI scores, medications, and duration of T2D as listed in Table 1 using the Cox proportional hazards regression. * p<0.05, ** p<0.01, *** p<0.001.

Table S8: Mortality Risk, Stratified by Variables, in Insulin-treated T2D Patients on GLP-1 RA vs. DPP-4 Inhibitors

	DPP-4 inhibitor users			GLP-1 RA users						
Variables	n	PY	IR	n	PY	IR	cHR	(95% CI)	aHR	(95% CI)
Sex										
Female	205	11083	18.50	88	11708	7.52	0.41	(0.32, 0.53)***	0.38	(0.30, 0.50)***
Male	314	12053	26.05	137	12942	10.59	0.41	(0.33, 0.50)***	0.37	(0.30, 0.46)***
Age, years										
20-60	295	17905	16.48	104	18962	5.48	0.34	(0.27, 0.42)***	0.35	(0.28, 0.43)***
61-100	224	5230	42.83	121	5689	21.27	0.50	(0.40, 0.63)***	0.42	(0.33, 0.53)***
Hypertension										
No	112	7913	14.15	49	8322	5.89	0.42	(0.30, 0.58)***	0.39	(0.27, 0.55)***
Yes	407	15223	26.74	176	16328	10.78	0.41	(0.34, 0.49)***	0.37	(0.31, 0.45)***
Dyslipidemia										
No	104	3593	28.94	54	3998	13.51	0.47	(0.34, 0.65)***	0.39	(0.28, 0.55)***
Yes	415	19543	21.24	171	20652	8.28	0.39	(0.33, 0.47)***	0.37	(0.31, 0.44)***
CCI										
≤1	309	19117	16.16	142	20152	7.05	0.44	(0.36, 0.54)***	0.43	(0.35, 0.52)***
>1	210	4019	52.25	83	4499	18.45	0.36	(0.28, 0.46)***	0.30	(0.23, 0.39)***
DCSI										
≤1	216	12797	16.88	87	13669	6.36	0.38	(0.30, 0.49)***	0.37	(0.29, 0.48)***
>1	303	10339	29.31	138	10981	12.57	0.43	(0.35, 0.53)***	0.39	(0.32, 0.48)***
Number of oral antidiabetic drugs										

0-3	79	5106	15.47	43	5809	7.40	0.48	(0.33, 0.70)***	0.38	(0.26, 0.56)***
>3	440	18029	24.40	182	18841	9.66	0.40	(0.34, 0.48)***	0.37	(0.31, 0.44)***
Statin										
No	117	5361	21.82	58	6091	9.52	0.45	(0.33, 0.62)***	0.39	(0.28, 0.55)***
Yes	402	17775	22.62	167	18559	9.00	0.40	(0.33, 0.48)***	0.37	(0.31, 0.45)***
Duration of T2D, years										
≤5	160	7957	20.11	54	7952	6.79	0.35	(0.26, 0.48)***	0.34	(0.25, 0.47)***
>5	359	15179	23.65	171	16698	10.24	0.43	(0.36, 0.52)***	0.39	(0.33, 0.47)***

Abbreviations: T2D, type 2 diabetes; GLP-1 RA, glucagon-like peptide-1 receptor agonists; DPP-4, dipeptidyl peptidase-4; PY: person-year, IR: incidence rate, per 1000 persons/years; cHR: crude hazard ratio; aHR: adjusted hazard ratio; CI: confidence interval. COPD, chronic obstructive pulmonary disease; CCI, Charlson Comorbidity Index; DCSI, Diabetes Complication Severity Index.

aHR: adjusted for age, sex, obesity, comorbidities, CCI, DCSI scores, medications, and duration of T2D as listed in Table 1 using the Cox proportional hazards regression. *** $p<0.001$.

Table S9: Hospitalization for CAD Risk, Stratified by Variables, in Insulin-treated T2D Patients on GLP-1 RA vs. Sulfonylurea

	Sulfonylurea users			GLP-1 RA users						
Variables	n	PY	IR	n	PY	IR	cHR	(95% CI)	aHR	(95% CI)
Sex										
Female	125	8345	14.98	90	9290	9.69	0.66	(0.50, 0.87)**	0.69	(0.53, 0.92)**
Male	139	8757	15.87	126	9340	13.49	0.86	(0.67, 1.09)	0.76	(0.59, 0.97)*
Age, years										
20-60	149	12603	11.82	118	13724	8.60	0.74	(0.58, 0.94)*	0.73	(0.57, 0.93)*
61-100	115	4498	25.57	98	4906	19.98	0.81	(0.62, 1.07)	0.75	(0.57, 0.99)*
Hypertension										
No	48	5523	8.69	40	6165	6.49	0.76	(0.50, 1.16)	0.75	(0.49, 1.16)
Yes	216	11579	18.65	176	12464	14.12	0.77	(0.63, 0.94)**	0.74	(0.60, 0.90)**
Dyslipidemia										
No	40	2917	13.71	21	3293	6.38	0.49	(0.29, 0.84)**	0.45	(0.26, 0.80)**
Yes	224	14185	15.79	195	15337	12.71	0.81	(0.67, 0.98)*	0.79	(0.65, 0.95)*
CCI										
≤1	200	13605	14.70	150	14975	10.02	0.70	(0.56, 0.86)***	0.68	(0.55, 0.84)***
>1	64	3497	18.30	66	3655	18.06	0.98	(0.70, 1.39)	0.92	(0.65, 1.31)
DCSI										
≤1	90	9170	9.81	52	9954	5.22	0.54	(0.38, 0.76)***	0.58	(0.41, 0.82)**
>1	174	7932	21.94	164	8676	18.90	0.88	(0.71, 1.09)	0.83	(0.67, 1.03)
Number of oral antidiabetic drugs										

0-3	62	4574	13.56	46	4816	9.55	0.72	(0.49, 1.06)	0.67	(0.45, 0.99)*
>3	202	12528	16.12	170	13814	12.31	0.77	(0.63, 0.94)*	0.76	(0.62, 0.93)**
Statin										
No	43	4383	9.81	35	4616	7.58	0.77	(0.49, 1.21)	0.71	(0.44, 1.13)
Yes	221	12719	17.38	181	14014	12.92	0.75	(0.62, 0.92)**	0.73	(0.60, 0.89)**
Duration of T2D, years										
≤5	72	6189	11.63	45	5847	7.70	0.71	(0.49, 1.04)	0.76	(0.52, 1.12)
>5	192	10913	17.59	171	12783	13.38	0.76	(0.61, 0.93)**	0.73	(0.59, 0.90)**

Abbreviations: CAD, coronary artery disease; T2D, type 2 diabetes; GLP-1 RA, glucagon-like peptide-1 receptor agonists; PY: person-year, IR: incidence rate, per 1000 persons/years; cHR: crude hazard ratio; aHR: adjusted hazard ratio; CI: confidence interval. COPD, chronic obstructive pulmonary disease; CCI, Charlson Comorbidity Index; DCSI, Diabetes Complication Severity Index.

aHR: adjusted for age, sex, obesity, comorbidities, CCI, DCSI scores, medications, and duration of T2D as listed in Supplementary Table 2 using the Cox proportional hazards regression. * $p<0.05$, ** $p<0.01$, *** $p<0.001$.

Table S10: Hospitalization for Stroke Risk, Stratified by Variables, in Insulin-treated T2D Patients on GLP-1 RA vs. Sulfonylurea

	Sulfonylurea users			GLP-1 RA users						
Variables	n	PY	IR	n	PY	IR	cHR	(95% CI)	aHR	(95% CI)
Sex										
Female	168	8292	20.26	106	9213	11.51	0.58	(0.45, 0.74)***	0.60	(0.47, 0.76)***
Male	157	8768	17.91	119	9384	12.68	0.72	(0.56, 0.91)**	0.68	(0.53, 0.87)**
Age, years										
20-60	171	12644	13.52	119	13698	8.69	0.66	(0.52, 0.83)***	0.65	(0.51, 0.83)***
61-100	154	4415	34.88	106	4900	21.63	0.63	(0.49, 0.80)***	0.60	(0.46, 0.77)***
Hypertension										
No	56	5540	10.11	44	6160	7.14	0.71	(0.48, 1.05)	0.71	(0.47, 1.06)
Yes	269	11520	23.35	181	12438	14.55	0.63	(0.52, 0.77)***	0.62	(0.52, 0.76)***
Dyslipidemia										
No	62	2880	21.53	43	3251	13.23	0.63	(0.43, 0.94)*	0.66	(0.44, 1.00)*
Yes	263	14179	18.55	182	15347	11.86	0.65	(0.53, 0.78)***	0.64	(0.53, 0.78)***
CCI										
≤1	218	13647	15.97	139	14966	9.29	0.59	(0.48, 0.73)***	0.58	(0.47, 0.72)***
>1	107	3412	31.36	86	3632	23.68	0.77	(0.58, 1.02)	0.73	(0.54, 0.98)*
DCSI										
≤1	115	9148	12.57	72	9889	7.28	0.59	(0.44, 0.80)***	0.59	(0.44, 0.80)***
>1	210	7911	26.54	153	8709	17.57	0.67	(0.54, 0.83)***	0.67	(0.54, 0.83)***
Number of oral antidiabetic drugs										

0-3	70	4611	15.18	44	4813	9.14	0.63	(0.43, 0.92)*	0.59	(0.40, 0.87)**
>3	255	12449	20.48	181	13785	13.13	0.65	(0.53, 0.78)***	0.65	(0.54, 0.79)***
Statin										
No	83	4324	19.20	49	4606	10.64	0.57	(0.40, 0.81)**	0.54	(0.37, 0.78)***
Yes	242	12736	19.00	176	13992	12.58	0.67	(0.55, 0.81)***	0.66	(0.54, 0.81)***
Duration of T2D, years										
≤5	74	6200	11.94	50	5846	8.55	0.76	(0.53, 1.09)	0.72	(0.49, 1.05)
>5	251	10860	23.11	175	12752	13.72	0.60	(0.49, 0.73)***	0.61	(0.50, 0.74)***

Abbreviations: T2D, type 2 diabetes; GLP-1 RA, glucagon-like peptide-1 receptor agonists; PY: person-year, IR: incidence rate, per 1000 persons/years; cHR: crude hazard ratio; aHR: adjusted hazard ratio; CI: confidence interval. COPD, chronic obstructive pulmonary disease; CCI, Charlson Comorbidity Index; DCSI, Diabetes Complication Severity Index.

aHR: adjusted for age, sex, obesity, comorbidities, CCI, DCSI scores, medications, and duration of T2D as listed in Supplementary Table 2 using the Cox proportional hazards regression. * p<0.05, ** p<0.01, *** p<0.001.

Table S11: Hospitalization for Heart Failure Risk, Stratified by Variables, in Insulin-treated T2D Patients on GLP-1 RA vs. Sulfonylurea

	Sulfonylurea users			GLP-1 RA users						
Variables	n	PY	IR	n	PY	IR	cHR	(95% CI)	aHR	(95% CI)
Sex										
Female	91	8468	10.75	41	9412	4.36	0.40	(0.28, 0.58)***	0.43	(0.30, 0.63)***
Male	69	8931	7.73	51	9492	5.37	0.72	(0.50, 1.04)	0.67	(0.46, 0.97)*
Age, years										
20-60	82	12773	6.42	43	13886	3.10	0.49	(0.34, 0.71)***	0.50	(0.34, 0.72)***
61-100	78	4626	16.86	49	5018	9.77	0.59	(0.41, 0.85)**	0.58	(0.40, 0.83)**
Hypertension										
No	23	5594	4.11	14	6225	2.25	0.55	(0.28, 1.07)	0.48	(0.24, 0.96)*
Yes	137	11805	11.61	78	12679	6.15	0.54	(0.41, 0.71)***	0.54	(0.41, 0.72)***
Dyslipidemia										
No	27	2952	9.14	13	3315	3.92	0.45	(0.23, 0.88)*	0.37	(0.18, 0.75)**
Yes	133	14446	9.21	79	15589	5.07	0.55	(0.42, 0.73)***	0.56	(0.42, 0.74)***
CCI										
≤1	110	13867	7.93	63	15165	4.15	0.53	(0.39, 0.72)***	0.52	(0.38, 0.72)***
>1	50	3532	14.16	29	3739	7.76	0.56	(0.35, 0.89)*	0.55	(0.34, 0.88)*
DCSI										
≤1	38	9280	4.09	25	10013	2.50	0.62	(0.37, 1.03)	0.65	(0.39, 1.09)
>1	122	8118	15.03	67	8891	7.54	0.51	(0.38, 0.68)***	0.53	(0.39, 0.71)***
Number of oral antidiabetic drugs										

0-3	29	4661	6.22	16	4877	3.28	0.55	(0.30, 1.01)	0.51	(0.27, 0.96)*
>3	131	12737	10.28	76	14027	5.42	0.53	(0.40, 0.70)***	0.56	(0.42, 0.74)***
Statin										
No	31	4418	7.02	15	4663	3.22	0.47	(0.25, 0.87)*	0.47	(0.25, 0.90)*
Yes	129	12981	9.94	77	14241	5.41	0.55	(0.41, 0.73)***	0.55	(0.41, 0.73)***
Duration of T2D, years										
≤5	46	6259	7.35	19	5919	3.21	0.48	(0.28, 0.82)**	0.53	(0.30, 0.94)*
>5	114	11140	10.23	73	12985	5.62	0.54	(0.41, 0.73)***	0.54	(0.40, 0.73)***

Abbreviations: T2D, type 2 diabetes; GLP-1 RA, glucagon-like peptide-1 receptor agonists; PY: person-year, IR: incidence rate, per 1000 persons/years; cHR: crude hazard ratio; aHR: adjusted hazard ratio; CI: confidence interval. COPD, chronic obstructive pulmonary disease; CCI, Charlson Comorbidity Index; DCSI, Diabetes Complication Severity Index.

aHR: adjusted for age, sex, obesity, comorbidities, CCI, DCSI scores, medications, and duration of T2D as listed in Supplementary Table 2 using the Cox proportional hazards regression. * p<0.05, ** p<0.01, *** p<0.001.

Table S12: ESKD Risk, Stratified by Variables, in Insulin-treated T2D Patients on GLP-1 RA vs. Sulfonylurea

	Sulfonylurea users			GLP-1 RA users						
Variables	n	PY	IR	n	PY	IR	cHR	(95% CI)	aHR	(95% CI)
Sex										
Female	42	8536	4.92	15	9444	1.59	0.33	(0.18, 0.59)***	0.32	(0.17, 0.59)***
Male	58	8942	6.49	24	9544	2.51	0.41	(0.25, 0.66)***	0.40	(0.25, 0.66)***
Age, years										
20-60	68	12806	5.31	23	13927	1.65	0.32	(0.20, 0.52)***	0.34	(0.21, 0.55)***
61-100	32	4673	6.85	16	5061	3.16	0.48	(0.26, 0.87)*	0.44	(0.22, 0.86)*
Hypertension										
No	7	5611	1.25	5	6245	0.80	0.70	(0.22, 2.25)	0.44	(0.11, 1.69)
Yes	93	11867	7.84	34	12743	2.67	0.35	(0.24, 0.52)***	0.37	(0.25, 0.56)***
Dyslipidemia										
No	21	2961	7.09	4	3335	1.20	0.18	(0.06, 0.53)**	0.20	(0.05, 0.73)*
Yes	79	14518	5.44	35	15653	2.24	0.42	(0.28, 0.63)***	0.42	(0.28, 0.64)***
CCI										
≤1	56	13962	4.01	18	15236	1.18	0.31	(0.18, 0.53)***	0.30	(0.17, 0.51)***
>1	44	3516	12.51	21	3752	5.60	0.45	(0.27, 0.76)**	0.44	(0.25, 0.78)**
DCSI										
≤1	19	9330	2.04	3	10053	0.30	0.17	(0.05, 0.58)**	0.14	(0.04, 0.48)**
>1	81	8148	9.94	36	8935	4.03	0.41	(0.28, 0.61)***	0.45	(0.30, 0.67)***
Number of oral antidiabetic drugs										

0-3	25	4703	5.32	8	4902	1.63	0.35	(0.16, 0.78)*	0.29	(0.12, 0.70)**
>3	75	12775	5.87	31	14086	2.20	0.38	(0.25, 0.57)***	0.43	(0.28, 0.67)***
Statin										
No	25	4429	5.64	5	4695	1.06	0.21	(0.08, 0.55)**	0.14	(0.05, 0.43)***
Yes	75	13049	5.75	34	14293	2.38	0.42	(0.28, 0.63)***	0.40	(0.26, 0.61)***
Duration of T2D, years										
≤5	24	6322	3.80	7	5942	1.18	0.36	(0.15, 0.83)*	0.48	(0.20, 1.20)
>5	76	11156	6.81	32	13046	2.45	0.36	(0.24, 0.54)***	0.39	(0.25, 0.59)***

Abbreviations: ESKD, end-stage kidney disease; T2D, type 2 diabetes; GLP-1 RA, glucagon-like peptide-1 receptor agonists; PY: person-year, IR: incidence rate, per 1000 persons/years; cHR: crude hazard ratio; aHR: adjusted hazard ratio; CI: confidence interval. COPD, chronic obstructive pulmonary disease; CCI, Charlson Comorbidity Index; DCSI, Diabetes Complication Severity Index.

aHR: adjusted for age, sex, obesity, comorbidities, CCI, DCSI scores, medications, and duration of T2D as listed in Supplementary Table 2 using the Cox proportional hazards regression. * $p<0.05$, ** $p<0.01$, *** $p<0.001$.

Table S13: Mortality Risk, Stratified by Variables, in Insulin-treated T2D Patients on GLP-1 RA vs. Sulfonylurea

	Sulfonylurea users			GLP-1 RA users						
Variables	n	PY	IR	n	PY	IR	cHR	(95% CI)	aHR	(95% CI)
Sex										
Female	198	8630	22.94	93	9476	9.81	0.43	(0.34, 0.55)***	0.47	(0.36, 0.60)***
Male	233	9065	25.70	117	9576	12.22	0.48	(0.38, 0.60)***	0.46	(0.36, 0.57)***
Age, years										
20-60	209	12975	16.11	88	13970	6.30	0.39	(0.31, 0.50)***	0.42	(0.33, 0.54)***
61-100	222	4720	47.03	122	5082	24.01	0.53	(0.42, 0.66)***	0.50	(0.40, 0.63)***
Hypertension										
No	92	5636	16.32	33	6253	5.28	0.33	(0.22, 0.49)***	0.29	(0.19, 0.43)***
Yes	339	12060	28.11	177	12799	13.83	0.50	(0.41, 0.59)***	0.50	(0.41, 0.60)***
Dyslipidemia										
No	107	3011	35.53	45	3343	13.46	0.37	(0.26, 0.53)***	0.36	(0.25, 0.52)***
Yes	324	14684	22.06	165	15709	10.50	0.48	(0.40, 0.58)***	0.49	(0.40, 0.59)***
CCI										
≤1	229	14086	16.26	122	15264	7.99	0.49	(0.40, 0.62)***	0.49	(0.40, 0.62)***
>1	202	3610	55.96	88	3788	23.23	0.42	(0.33, 0.54)***	0.43	(0.33, 0.56)***
DCSI										
≤1	154	9364	16.45	74	10057	7.36	0.45	(0.34, 0.60)***	0.45	(0.34, 0.60)***
>1	277	8331	33.25	136	8995	15.12	0.46	(0.37, 0.56)***	0.45	(0.37, 0.56)***

Number of oral antidiabetic drugs										
0-3	82	4762	17.22	47	4912	9.57	0.57	(0.40, 0.82)**	0.45	(0.30, 0.66)***
>3	349	12933	26.98	163	14140	11.53	0.43	(0.36, 0.52)***	0.46	(0.38, 0.56)***
Statin										
No	127	4476	28.38	51	4704	10.84	0.38	(0.28, 0.53)***	0.33	(0.23, 0.46)***
Yes	304	13220	23.00	159	14348	11.08	0.49	(0.40, 0.59)***	0.49	(0.41, 0.60)***
Duration of T2D, years										
≤5	120	6377	18.82	50	5953	8.40	0.45	(0.32, 0.63)***	0.42	(0.30, 0.60)***
>5	311	11318	27.48	160	13099	12.22	0.45	(0.37, 0.55)***	0.45	(0.37, 0.55)***

Abbreviations: T2D, type 2 diabetes; GLP-1 RA, glucagon-like peptide-1 receptor agonists; PY: person-year, IR: incidence rate, per 1000 persons/years; cHR: crude hazard ratio; aHR: adjusted hazard ratio; CI: confidence interval. COPD, chronic obstructive pulmonary disease; CCI, Charlson Comorbidity Index; DCSI, Diabetes Complication Severity Index.

aHR: adjusted for age, sex, obesity, comorbidities, CCI, DCSI scores, medications, and duration of T2D as listed in Supplementary Table 2 using the Cox proportional hazards regression. ** p<0.01, *** p<0.001.

Table S14: Diseases and associated ICD-9 and ICD-10 codes

Disease	ICD-9-CM codes	ICD-10-CM codes
Alcohol-related disorder	291, 303, 305.0, 357.5, 425.5, 535.3, 571.0-571.3, 790.3, 980.0, V11.3, V79.1	G31.2, G72.1, G62.1, E24.4, F10, I42.6, K29.2, K70, K85.2, K86.0, O99.31, R78.0, T51.0X1A-T51.0X4A, Z71.41
Atrial fibrillation	427	I46.2, I46.8, I46.9, I47-I49, R00.1
Coronary artery disease	410-414	I20-I22, I24, I25
Chronic obstructive pulmonary disease	491, 492, or 496	J41, J42, J44, J43
Cancers	140-189, 190-199, 200-209, 210-239	C00-C63, C64-C80, C7A-C7B, C81-C96, D37, D38, D39, D40, D41, D42, D43, D44, D45, D46, D47, D48, D49
Chronic kidney disease	585-586, 593.9	N18, N19, N28.9, N29
Dialysis	V56.0, V56.8, V45.1	Z49.31, Z49.32, Z99.2
Kidney transplant	V42.0	Z94.0
Diabetic retinopathy	362.0、362.1 and ICD-9-PCS 142.9, 143, 145, 147.2	E08.31, E08.32, E08.33, E08.34, E08.35, E09.31, E09.32, E09.33, E09.34, E09.35, E11.31-E11.34、E11.35、E13.31, E13.32, E13.33, E13.34, E13.35、H35.0 及 ICD-10-PCS 08943ZZ, 08BE3ZZ, 08BF3ZZ, 08QE, 08QF
Dyslipidemia	272	E71.30, E75.21, E75.22, E75.24, E75.3, E75.5, E75.6, E77, E78.0, E78.1, E78.2, E78.3, E78.4, E78.5, E78.6, E78.70, E78.79, E78.8, E78.9
Hypertension	401–405	I10, I11, I12, I13, I15, N26.2
Heart failure	428	I50
Liver cirrhosis	571.5, 571.2, 571.6	K70.2, K70.30, K70.31, K74.0, K74.1, K74.2, K74.60, K74.69, K74.3, K74.4, K74.5

Leg amputation	V49.71–V49.77 or V52.1, ICD-9-PCS 84.11–84.17	Z89.4, Z89.5, Z89.6, ICD-10-PCS 0Y6
Obesity	278.0, 278.1, 783.1	R63.5, E65, E66.09, E66.1, E66.8, E66.9, E66.01, E66.2
Peripheral artery disease	440.0, 440.20, 440.21, 440.22, 440.23, 440.24, 440.29, 440.3, 443.9, 443.81, 443.89	I70.0, I70.2, I70.3, I70.4, I70.5, I70.6, I70.7, I70.92, I75.0, I73.8, I73.9, I79.1, I79.8
Smoking status	305.1, 989.84, V15.82	F17.2, O99.33, T65.211A-T65.214A, T65.221A-T65.224A, T65.291A-T65.294A, Z71.6, Z72.0, Z87.891
Stroke	430-438	G45.0, G45.1, G45.2, G45.3, G45.4, G45.8, G45.9, G46, I60, I61, I62, I63, I65, I66, I67.0, I67.1, I67.2, I67.3, I67.4, I67.5, I67.6, I67.7, I67.8, I67.9, I68, I69
Type 2 diabetes mellitus	250.x0, 250.x2	E11
Type 1 diabetes mellitus	250.x1, 250.x3	E10
Visual loss	369, V41	H54

Supplementary Figure Legends

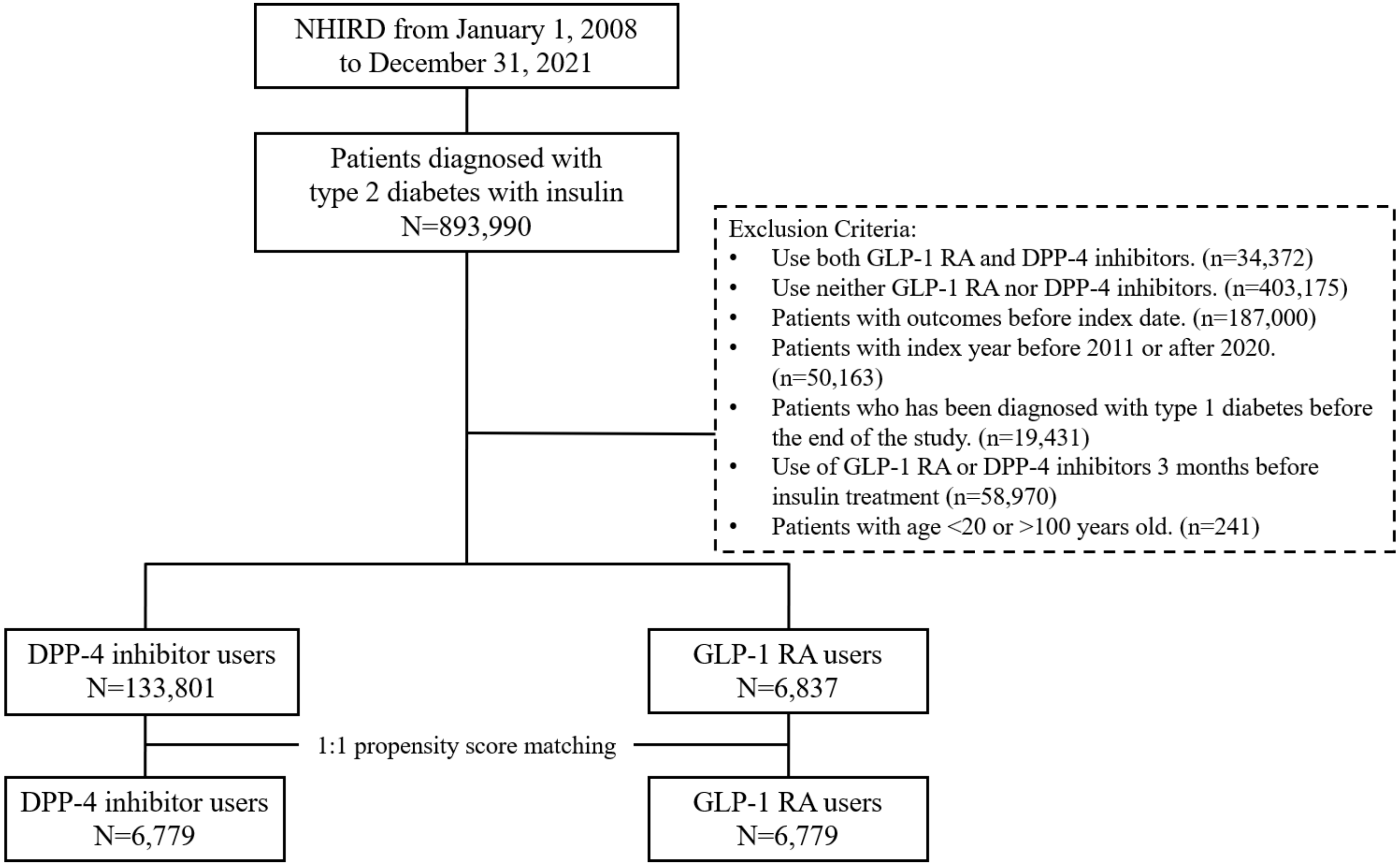

Figure S1 Patient Selection Flowchart for the Use of GLP-1 RA and DPP-4 Inhibitors

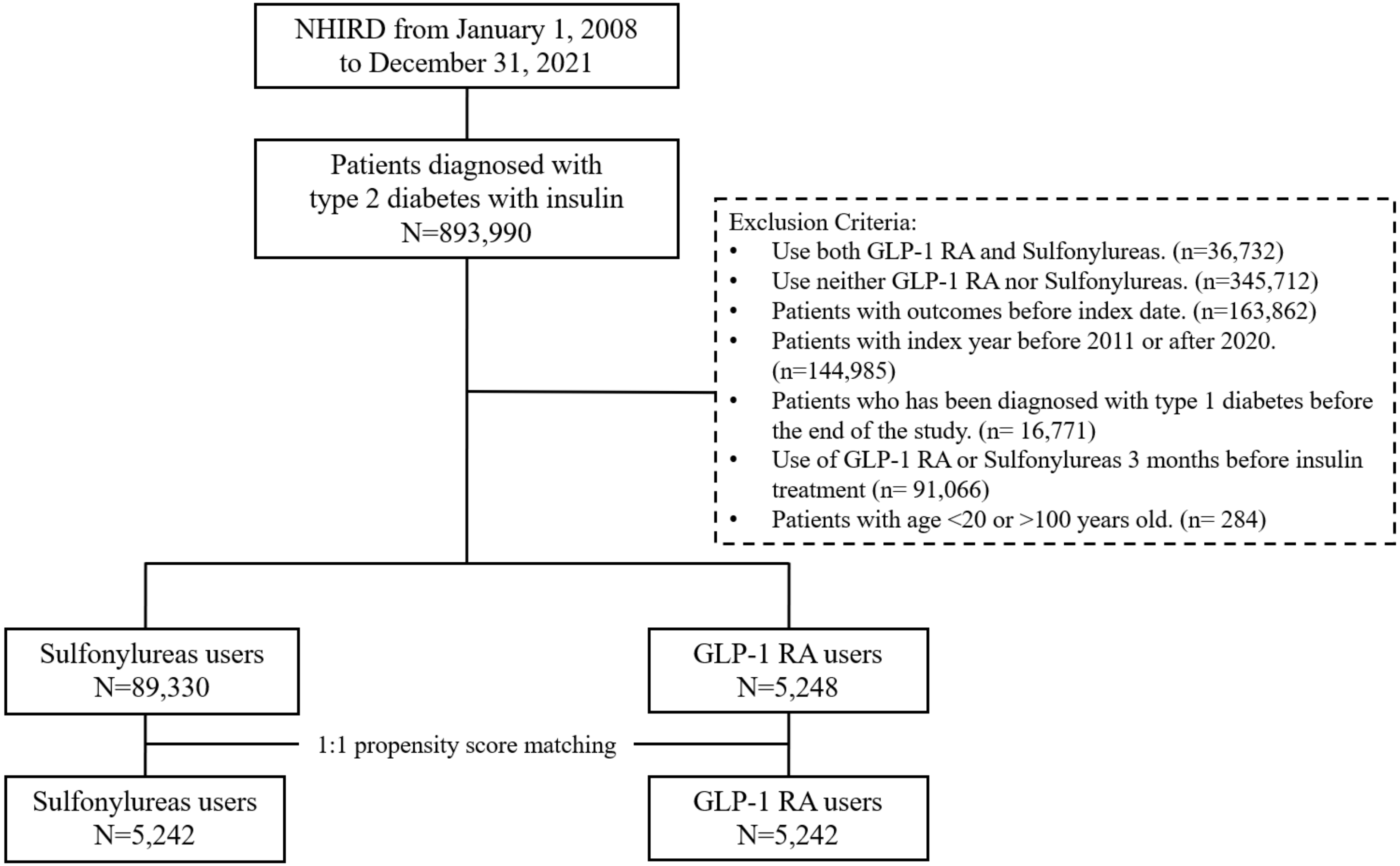

Figure S2 Patient Selection Flowchart for the Use of GLP-1 RA and Sulfonylureas

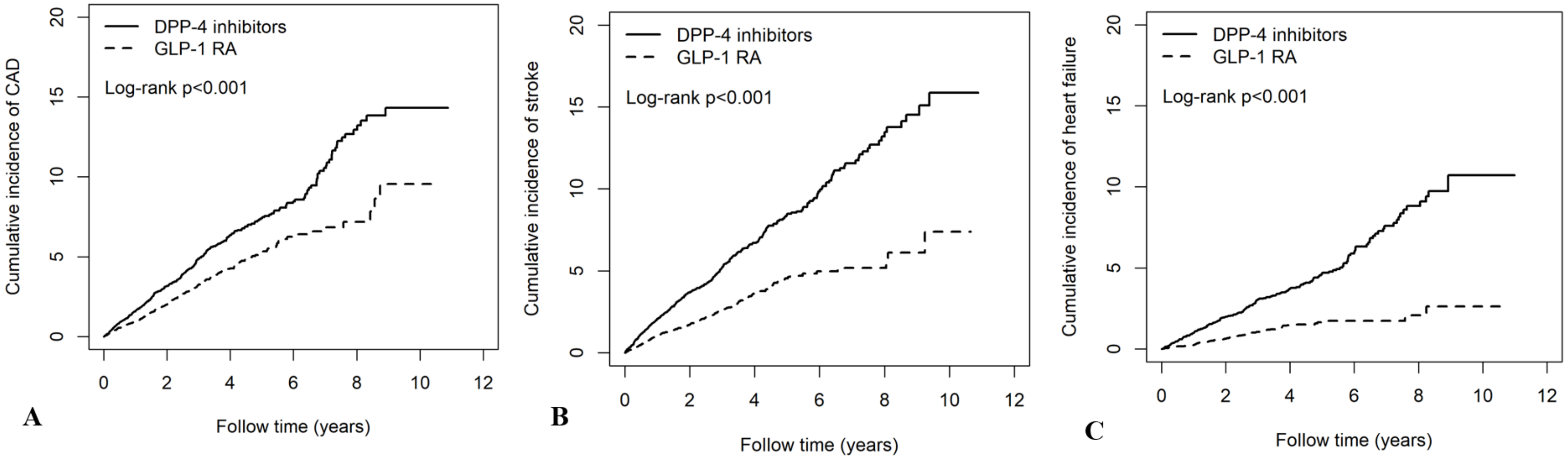

Figure S3 Cumulative incidence of hospitalization for (A) coronary artery disease (CAD), (B) stroke, and (C) heart failure in GLP-1 RA and DPP-4 inhibitor users

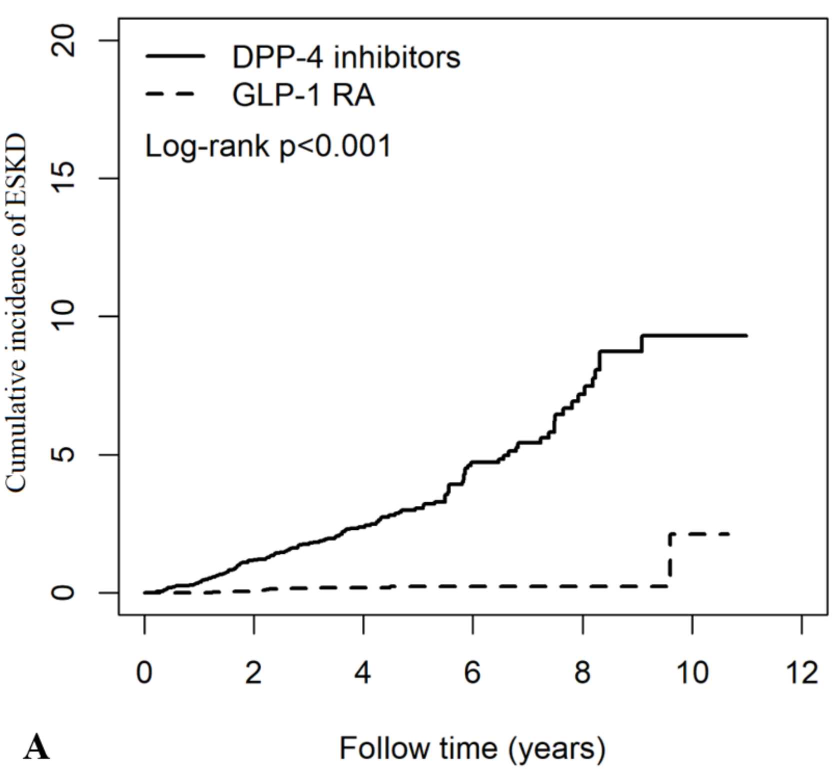

A

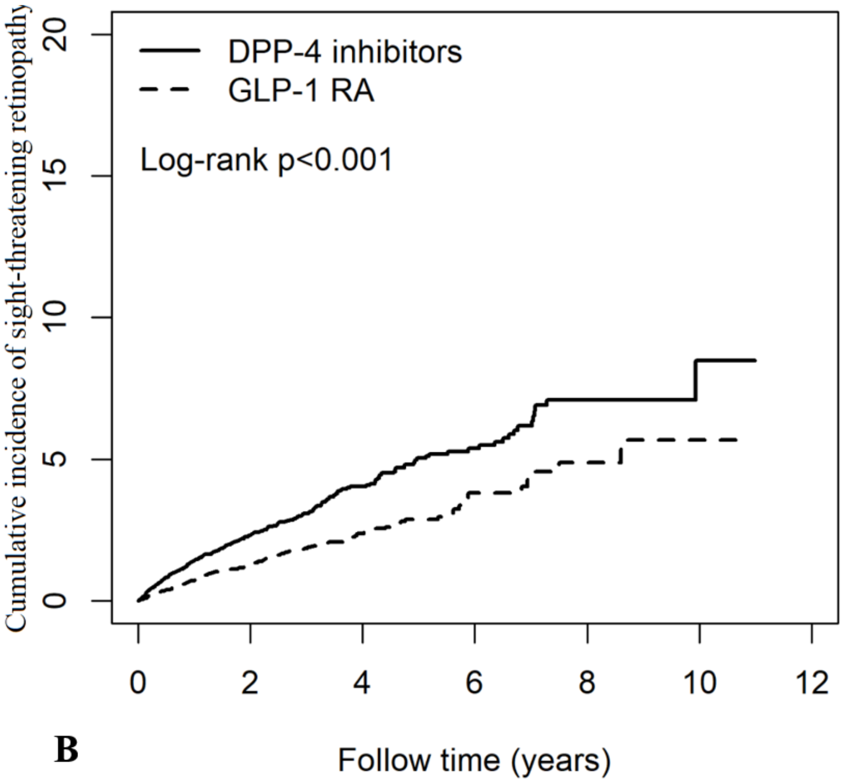

B

Figure S4 Cumulative incidence of (A) ESKD, (B) sight-threatening retinopathy in GLP-1 RA and DPP-4 inhibitor users

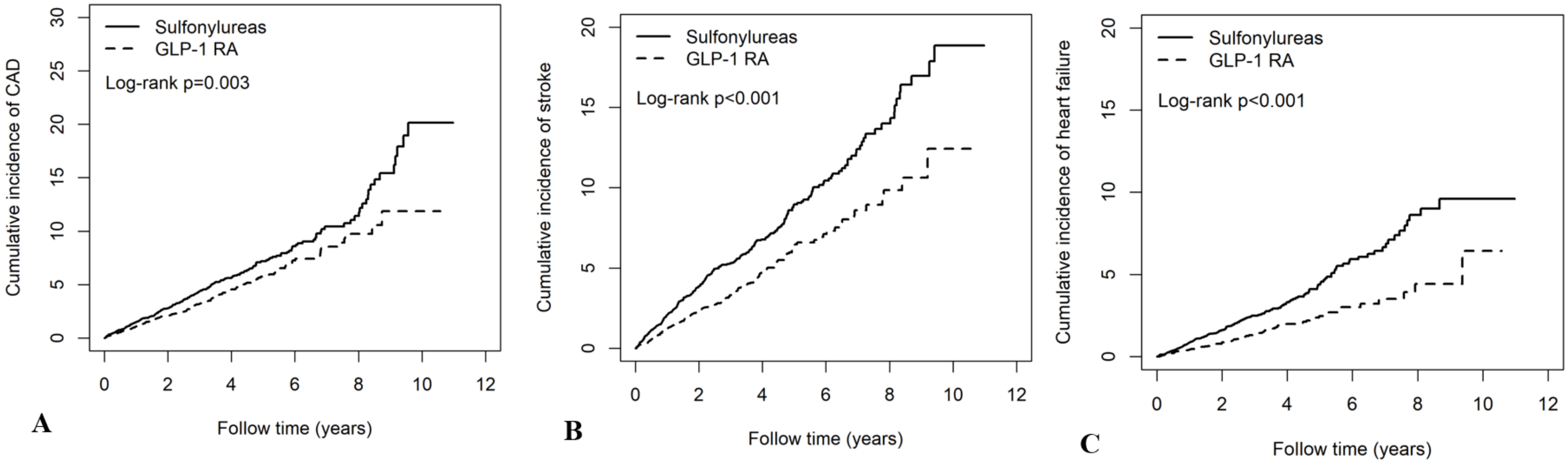

Figure S5 Cumulative incidence of hospitalization for (A) coronary artery disease (CAD), (B) stroke, and (C) heart failure in GLP-1 RA and sulfonylurea users

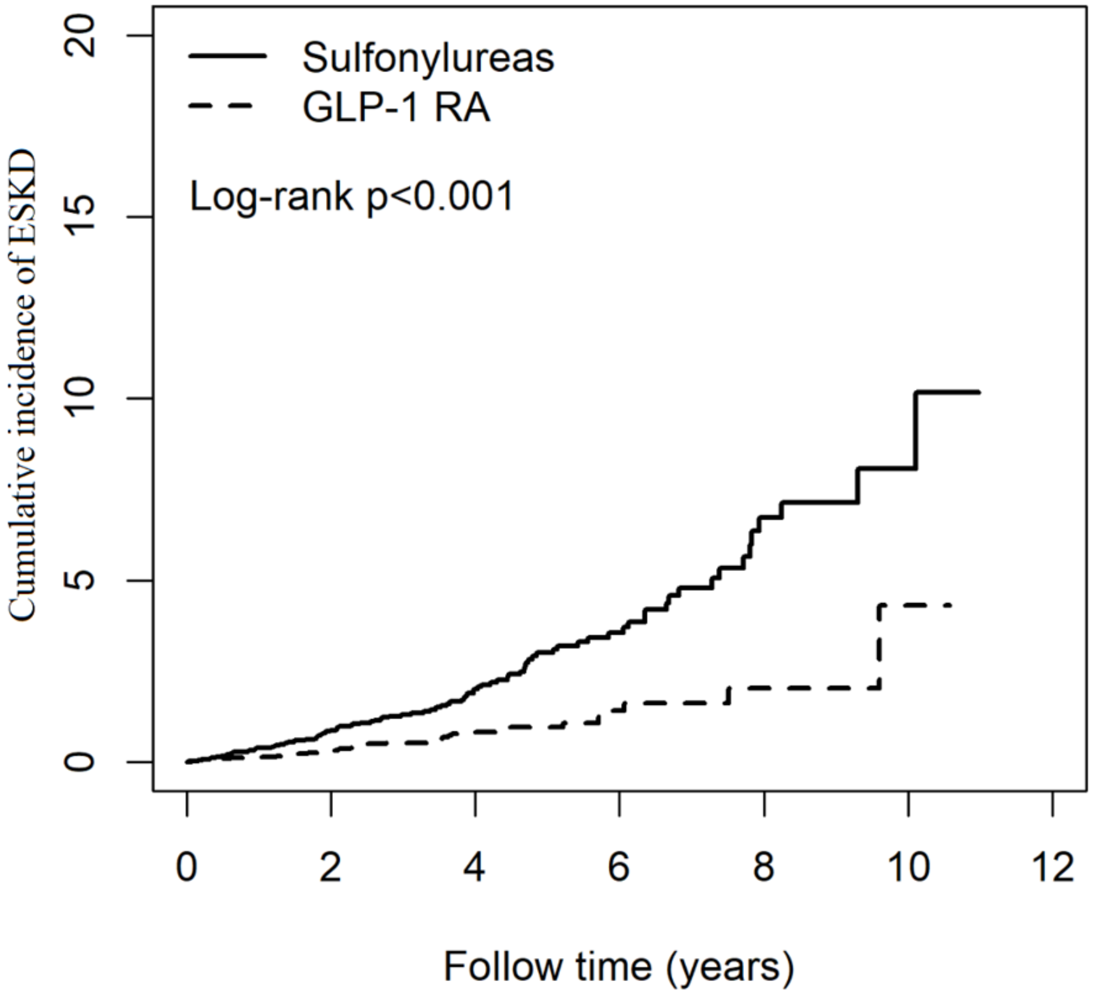

Figure S6 Cumulative incidence of end-stage kidney disease (ESKD) in GLP-1 RA and sulfonylurea users
